# Phylo-geo-network and haplogroup analysis of 611 novel coronavirus (SARS-CoV-2) genomes from India

**DOI:** 10.26508/lsa.202000925

**Published:** 2021-03-16

**Authors:** Rezwanuzzaman Laskar, Safdar Ali

**Affiliations:** Clinical and Applied Genomics Laboratory, Department of Biological Sciences, Aliah University, Kolkata, India

## Abstract

The novel coronavirus (SARS-CoV-2) from Wuhan China discovered in December 2019 has since developed into a global epidemic. Presently, we constructed and analyzed the phylo-geo-network of SARS-CoV-2 genomes from across India to understand the viral evolution in the country. A total of 611 full-length genomes from different states of India were extracted from the EpiCov repository of GISAID initiative on 6 June, 2020. Their alignment with the reference sequence (Wuhan, NCBI accession number NC_045512.2) uncovered 270 parsimony informative sites. Furthermore, 339 genomes were divided into 51 haplogroups. The network revealed the core haplogroup as that of reference sequence NC_045512.2 (Haplogroup A1) with 157 identical sequences present across 16 states. Remaining haplogroups had <10 identical sequences across a maximum of three states. Some states with fewer samples had more haplogroups. Forty-one haplogroups were localized exclusively to any one state. The two most common lineages are B6 and B1 (Pangolin) whereas clade A2a (Covidex) appears to be the most predominant in India. Because the pandemic is still emerging, the observations need to be monitored.

## Abstract (precis)

Evolution of SARS-CoV-2 in India across 51 haplogroups based on 152 parsimony informative sites revealed B6 and B1 (Pangolin) and A2a (Covidex) as the most prevalent lineage and clade, respectively.

## Introduction

Coronaviruses belonging to the family Coronaviridae have been named so owing to the resemblance of the virion electron microscopic structure to that of a crown wherein the spikes present on the virion surface provide for the crown-like similarity (Lin et al, 2005). Their genome has a positive single strand RNA of 26–32 kb in length and are known to infect a wide range of hosts (Lai & Cavanagh, 1997; Ismail et al, 2003; Weiss & Navas-Martin, 2005; Cavanagh, 2007; Su et al, 2016).

The novel coronavirus SARS-CoV-2 from Wuhan, China, was discovered in December 2019. Since its emergence, it has developed into a global epidemic (Rothan & Byrareddy, 2020). As of 22 January, 2021, there were 10,625,428 cases and 1,53,032 deaths in India due to SARS-CoV-2 (https://www.mygov.in/covid-19). At the same time, as per World Health Organization there have been 96,012,792 confirmed cases including 2,075,870 deaths worldwide due to COVID-19 (covid19.who.int). The SARS-CoV-2 is different from earlier coronavirus outbreaks, severe acute respiratory syndrome (SARS) coronavirus in 2002 and Middle East respiratory syndrome coronavirus in 2012 predominantly due to its extremely high transmission rates (Peiris et al, 2004; Zaki et al, 2012; Sun et al, 2020). The patients infected with SARS-CoV-2 have been observed to have varied symptoms ranging from normal flu like symptoms to high fever to invasive lesions (Chan et al, 2020; Huang et al, 2020; Zhu et al, 2020).

The SARS-CoV-2 belongs to genus betacoronavirus and sub-genus sarbecovirus with possible origin in bats supported by its similarity to two bat-derived coronavirus strains, bat-SL-CoVZC45 and bat-SL-CoVZXC21 (Lu et al, 2020; Zhou et al, 2020). Also, the ever-increasing number of people being infected globally provides for the most conducive environments for the virus to evolve. The availability of full genome sequences for SARS-CoV-2 in Global Initiative on Sharing All Influenza Data (GISAID) has aided the study of these evolving sequences with both global and local perspectives (Shu & McCauley, 2017).

At present, we build and analyze the phylo-geo-network of SARS-CoV-2 based on the publicly available full-length sequences of SARS-CoV-2 from India. We also performed the haplogroup analysis with their defining mutations and phylogenetic lineage study along with geographical distributions. The present study would help us understand the evolutionary path of the virus in India, which would be an asset to counter the global burden of SARS-CoV-2 in future.

## Results

### Phylogenetic network analysis

**Figure 1.**
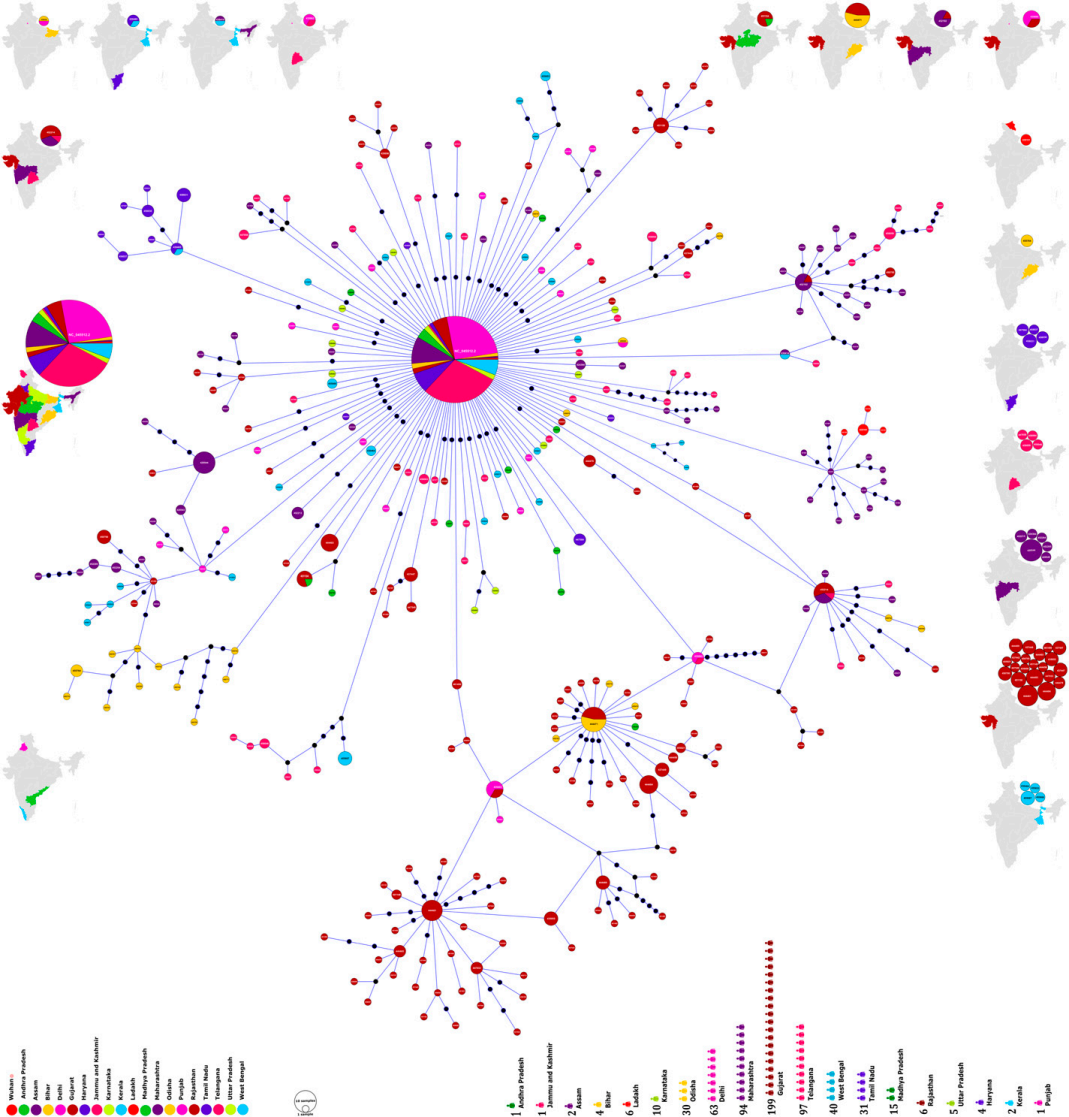
Phylogenomic geographic (phylo-geo) network of SARS-CoV-2 genomes from India. The nodes represented by circles have been named after the Accession Numbers of the defining sequences representing a particular cluster. The diameter of the circle corresponds to the number of sequences present therein. Thus, a bigger circle will imply more sequences. The different states of India have been represented by color coding and the number of sequences from each state used in the study has been shown in the lower panel of the figure. The distribution of haplogroups across different states is shown in the maps on the periphery such that haplogroups present only in one state are in the maps on the right side. Maps on other sides include haplogroups present in more than one state. Maps have been generated and powered by Bing (Geo Names; Microsoft, TomTom) through MS Excel 2019.

The alignment of 611 SARS-CoV-2 genomes and their subsequent analysis revealed a total of 493 segregating sites of which 270 were parsimony informative (PI) sites. The incidence of sites and their distribution across gaps and ambiguous sequences and statistical evaluation has been summarized in Table 1. A negative value of Tajimas D statistic suggests the significance of these sites in evolution of these genomes. The reported phylo-geo-network herein has been built using the 152 PI sites obtained after excluding the gaps and ambiguous sequences. The phylo-geo-network analysis of the studied genomes has been represented in Fig 1.

**Table 1.** Some key statistical parameters observed in the study.

S No	Network type	Number of segregating sites	Number of parsimony-informative sites		Nucleotide diversity	Tajima’s D statistic
			Excluding^a^	Including^a^		
1	Transitive consistency score	493	152	270	π = 0.00120683	D = −1.82662 p (D axis −1.82662) = 0.982906

a Gaps and ambiguous/missing (details in Table S3).

Interestingly, there was one sequence with genome id 458080 from Telangana, which was 100 percent identical to the Wuhan reference sequence (Tables S1 and S4). Although the absence of travel history for most of the studied patients and the sequences only being a partial representation of the total patients present makes the conclusion subjective, it does indicate about arrival of the virus directly from China to India.

Table S1 Details of SARS-CoV-2 genomes used in the study.

### Haplogroup analysis and distribution

**Figure 2.**
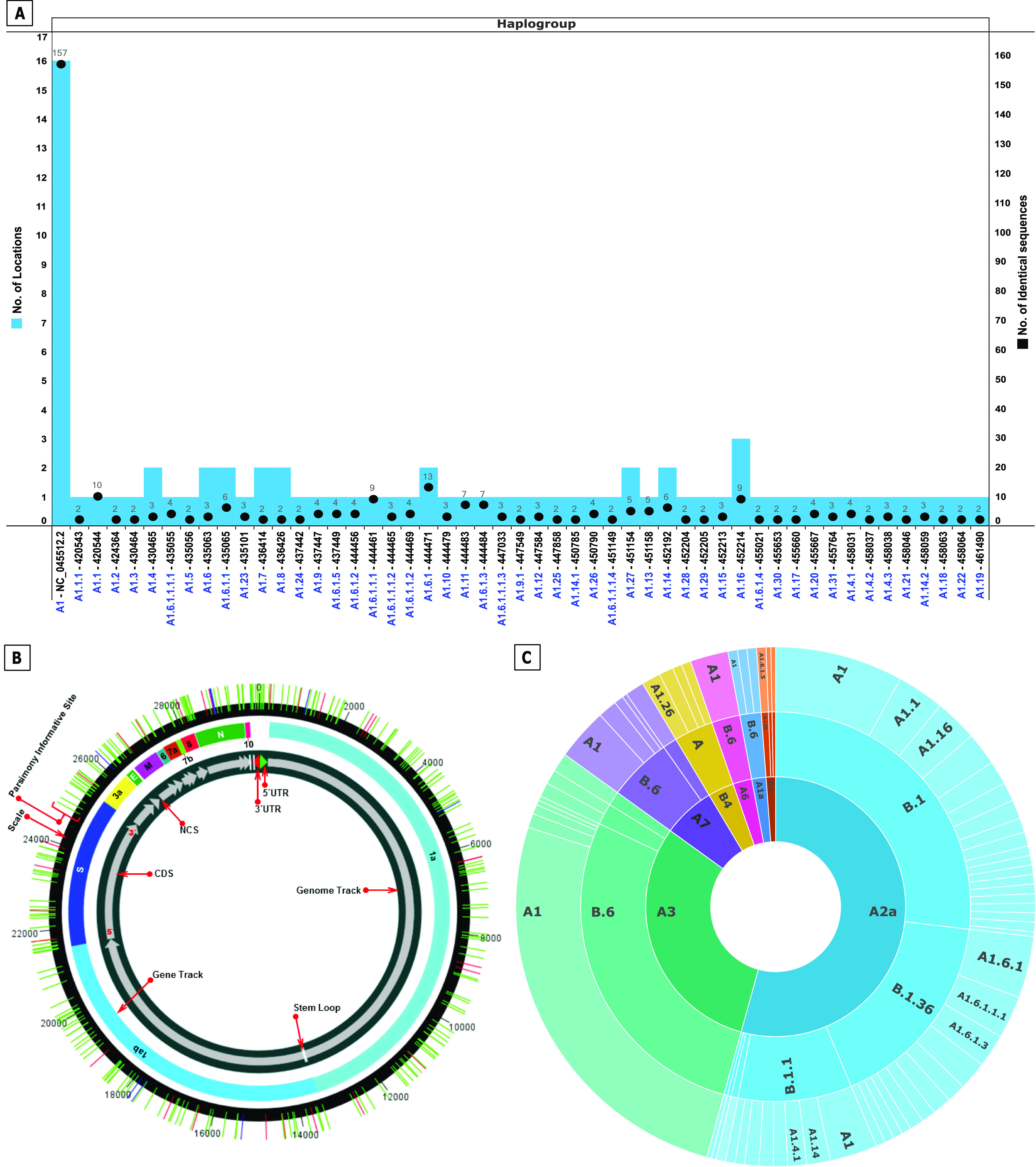
**Haplogroup distribution and **l**ineage analysis of studied genomes. (A)** Prevalence and geographical distribution of 51 haplogroups of SARS-CoV-2 genomes in India. The haplogroups are shown on the x-axis. The number of identical sequences present in a haplogroup is shown as bar whereas number of states, wherein the haplogroup is present is shown as a black dot. Note the maximum prevalence (157 sequences) and widespread distribution (16 states) of NC_045512.2 containing haplogroup (A1). For details of haplogroup IDs, identical sequences, and locations, please refer Table S2. **(B)** Distribution of parsimony informative sites across the SARS-CoV-2 genomes. The SARS-CoV-2 genome has been represented circularly along with the locations of different genes/ORFs/Non coding regions. Parsimony informative sites are shown as lines traversing the circle. **(C)** Lineage and Subtype Analysis of SARS-CoV-2 genomes in India. The outermost circle represents haplogroups reported in the study whereas the middle circle depicts lineage prediction by Pangolin Web. The innermost circle is the clade analysis by Covidex Web tool.

The network tree construction was accompanied by haplogroup determination of the studied genomes. The nodes representing haplogroups in phylo-geo-network in Fig 1 have been named as per accession number of the sequence defining the haplogroup. The nodal haplogroup represented by the Wuhan reference sequence NC_045512.2 has two maxima associated with it. The node has 157 sequences distributed across 16 states. The details of distribution of all identical sequences have been summarized in Fig 2A and Table S2.

Table S2 Details of identical sequences in the study and their geographical distribution.

We propose the nomenclature of the 51 observed haplogroups as per the path used to construct the network which has been explained with a couple of examples as follows. The haplogroup having NC_045512.2 was named A1 as the core of the network. From this cluster, many haplogroups emerged and so on. The haplogroup A1.1 (420544) is defined by five positions; 241 (C → T), 3037 (C → T), 4809 (C → T), 14408 (C → T), and 23403 (A → G). However, as we move to haplogroup A1.1.1 (420543), in addition to the above mutations, another one at position 8782 (C → T) is present which becomes the defining polymorphism for this haplogroup. Similarly, haplogroup A1.6 (435063) is defined by positions 241(C → T), 1059 (C → T), 3037 (C → T), 14408 (C → T), 23403 (A → G), and 25563 (G → T). Subsequently haplogroup A1.6.1 (444471) is characterized by mutation at positions 18877 (C → T) and 26735 (C → T) and haplogroup A1.6.1.1 by additional mutations at 22444 (C → T) and 28854 (C → T). The haplogroup lineage thus defined clearly indicates that A1 is the most prevalent one, whereas A1.6 is the most evolving one as it has the maximum number of steps going up to A1.6.1.1.1.4 reflective of five steps and stages of mutations/PI sites. The position of all the observed PI sites has been listed in Table 2 and Fig 2B and their details are summarized in Table S3. The haplogroup nomenclature has been listed in correlation with their genome IDs and location in Table 3. The observed PI sites reported in the study include most of the commonly reported sites from across the world besides some novel ones. However, we are not emphasizing on the novelty of sites because of the fast-changing scenario and rapidly emerging data.

Table S3 Details of parsimony informative sites including gaps and ambiguous sequences observed in the study.

**Table 2.** Distribution of parsimony informative sites across the genome of nCOV-2019.

S No	Genome region	Start position	End position	Size (bp)	No of parsimony sites	Strike-rate of parsimony sites^a^	Position of parsimony informative sites including the gaps and ambiguous sequences
1	5′UTR	1	265	265	9	29.4	22, 55, 56, 94, 106, 218, 219, 222, 241
2	*ORF1a*	266	13,483	13,218	100	132.2	506, 635, 771, 875, 884, 1059, 1094, 1191, 1218, 1281, 1397, 1589, 1599, 1707, 1820, 1846, 2143, 2368, 2480, 2558, 2632, 2836, 3037, 3039, 3054, 3085, 3426, 3472, 3634, 3686, 3737, 3817, 4067, 4084, 4255, 4354, 4372, 4444, 4679, 4809, 4866, 4893, 4965, 5029, 5062, 5139, 5572, 5700, 5826, 6081, 6310, 6312, 6402, 6466, 6541, 6573, 6616, 6868, 6989, 7319, 7392, 7600, 7945, 8022, 8026, 8080, 8296, 8460, 8653, 8782, 8917, 8950, 9389, 9438, 9628, 9693, 10138, 10277, 10369, 10478, 10479, 10679, 10702, 10771, 10815, 11074, 11083, 11200, 11306, 11335, 11457, 11572, 11620, 12076, 12439, 12616, 12685, 12757, 13458
3	*ORF1b*	13,468	21,555	8,088	58	139.4	13585, 13617, 13730, 13859, 14130, 14181, 14274, 14408, 14425, 14673, 14805, 15324, 15435, 15451, 15708, 16017, 16078, 16355, 16393, 16626, 16738, 16852, 16887, 16993, 17135, 17440, 17722, 17747, 17858, 17959, 18052, 18129, 18380, 18395, 18457, 18486, 18511, 18877, 19086, 19185, 19344, 19417, 19524, 19679, 19684, 19816, 19872, 19983, 20006, 20063, 20087, 20151, 20355, 20773, 21004, 21137, 21550, 21551
4	*S*	21,563	25,384	3,822	33	115.8	21575, 21627, 21628, 21646, 21724, 21792, 21795, 21890, 22289, 22343, 22374, 22444, 22468, 22530, 22663, 23120, 23236, 23277, 23111, 23403, 23593, 23638, 23678, 23815, 23821, 23929, 24811, 24933, 25098, 25290, 25314, 25381
5	*ORF3a*	25,393	26,220	828	10	82.8	25445, 25461, 25513, 25528, 25563, 25596, 25613, 25855, 25904, 26144
6	Non-coding	26,221	26,244	24	1	24	26226
7	*E*	26,245	26,472	228	5	45.6	26330, 26338, 26375, 26376, 26467
8	*M*	26,523	27,191	669	6	111.5	26530, 26681, 26730, 26735, 27110, 27191
9	*ORF6*	27,202	27,387	186	5	37.2	27213, 27379, 27382, 27383, 27384
10	*ORF7a*	27,394	27,759	366	1	366	27613
11	*ORF7b*	27,756	27,887	132	1	132	27874
12	Non-coding	27,888	27,893	6	1	6	27889
13	*ORF8*	27,894	28,259	366	7	52.3	28001, 28077, 28083, 28114, 28221, 28253, 28254
14	*N*	28,274	29,533	1,260	20	63	28289, 28311, 28312, 28326, 28371, 28396, 28688, 28795, 28854, 28878, 28881, 28882, 28883, 28948, 29039, 29188, 29197, 29236, 29451, 29474
15	Non-coding	29,534	29,557	24	3	8	29543, 29555, 29557
16	*ORF10*	29,558	29,674	117	0		
17	3′UTR	29,675	29,903	229	10	22.9	29722, 29734, 29742, 29743, 29774, 29827, 29829, 29830, 29870, 29874
	Total				270		

a Calculated by size/no. of parsimony sites in the region.

**Table 3.** Details of haplogroups: geographical distribution and phylogenetic lineage.

Haplogroup	Node label/Genome ID	State	Most common countries	Lineage analysis (Rambaut et al, 2020 *Preprint*)	Subtype analysis-SARS Cov 2 nextstrain (Hadfield et al, 2018)
Proposed	Assigned by (GISAID/NCBI)	Assigned by Database (GISAID/NCBI)	Assigned by Pangolin Web server	Prediction by Pangolin Web server	Prediction by Covidex Web server
A1	NC_045512.2	1. Assam	1. Australia, Singapore, USA	1. B	1. A1a
		2. Bihar	2. India, Singapore, Australia	2. B.1	2. A2
		3. Delhi	3. UK, Australia, USA	3. B.1.1	3. A2a
		4. Gujarat	4. UK, China, USA	4. B.1.5	4. A3
		5. Haryana	5. UK, Spain, Australia	5. B.6	5. A6
		6. Jammu	6. UK, USA, Australia		6. A7
		7. Karnataka	7. UK, USA, China		
		8. Madhya Pradesh			
		9. Maharashtra			
		10. Odisha			
		11. Rajasthan			
		12. Tamil Nadu			
		13. Telangana			
		14. Uttar Pradesh			
		15. West Bengal			
		16. Wuhan, China			
A1.1	420544	Maharashtra	UK, USA, Australia	B.1	A2a
A1.1.1	420543	Maharashtra	UK, USA, Australia	B.1	A2a
A1.10	444479	Gujarat	UK, USA, Australia	B.1	A2a
A1.11	444483	Gujarat	UK, USA, Australia	B.1	A2a
A1.12	447584	Tamil Nadu	India, Singapore, Australia	B.6	A3
A1.13	451158	Gujarat	UK, USA, Australia	B.1	A2a
A1.14	452192	1. Gujarat	UK, Australia, USA	1. B.1	A2a
		2. Maharashtra	UK, USA, Australia	2. B.1.1	
A1.14.1	450785	Gujarat	UK, USA, Australia	B.1	A2a
A1.14.2	458059	Telangana	UK, Australia, USA	B.1.1	A2a
A1.15	452213	Maharashtra	Australia, UK, Turkey	B.4	A3
A1.16	452214	1. Gujarat	UK, USA, Australia	B.1	A2a
		2. Maharashtra			
		3. Telangana			
A1.17	455660	West Bengal	UK, USA, Australia	B.1	A2a
A1.18	458063	Telangana	India, Singapore, Australia	B.6	A7
A1.19	461490	Gujarat	UK, USA, Australia	B.1	A2a
A1.2	424364	Maharashtra	UK, USA, Australia	B.1	A2a
A1.20	455667	West Bengal	UK, USA, Australia	B.1	A2a
A1.21	458046	Telangana	UK, Australia, Gambia	B.1.1.8	A2a
A1.22	458064	Telangana	UK, USA, Australia	B.1	A2a
A1.23	435101	Ladakh	Australia, UK, Turkey	B.4	A3
A1.24	437442	Gujarat	Australia, Singapore, USA	B.6	A1a
A1.25	447858	Telangana	India, Singapore, Australia	B.6	A3
A1.26	450790	Gujarat	China, South Korea, USA	A	B4
A1.27	451154	1. Gujarat	Australia, Singapore, USA	B.6	1. A3
		2. Madhya Pradesh			2. A7
			India, Singapore, Australia		
A1.28	452204	Maharashtra	China, South Korea, USA	A	B4
A1.29	452205	Maharashtra	China, South Korea, USA	A	B4
A1.3	430464	West Bengal	UK, Australia, USA	B.1.1	A2a
A1.30	455653	West Bengal	UK, USA, Australia	B.1	A2a
A1.31	455764	Odisha	China, South Korea, USA	A	B4
A1.4	430465	1. Tamil Nadu	UK, Australia, USA	B.1.1	A2a
		2. West Bengal			
A1.4.1	458031	Tamil Nadu	UK, Australia, USA	B.1.1	A2a
A1.4.2	458037	Tamil Nadu	UK, Australia, USA	B.1.1	A2a
A1.4.3	458038	Tamil Nadu	UK, Australia, USA	B.1.1	A2a
A1.5	435056	Gujarat	UK, USA, Australia	B.1	A2a
A1.6	435063	1. Delhi	UK, USA, Australia	B.1	A2a
		2. Telangana			
A1.6.1	444471	1. Gujarat	Saudi Arabia, UK, Turkey	B.1.36	A2a
		2. Odisha	Turkey, Finland, UK		
A1.6.1.1	435065	1. Delhi	Saudi Arabia, UK, Turkey	B.1.36	A2a
		2. Gujarat	Turkey, Finland, UK		
A1.6.1.1.1	444461	Gujarat	Saudi Arabia, UK, Turkey	B.1.36	A2a
			Turkey, Finland, UK		
A1.6.1.1.1.1	435055	Gujarat	Turkey, Finland, UK	B.1.36	A2a
A1.6.1.1.1.2	444465	Gujarat	Turkey, Finland, UK	B.1.36	A2a
A1.6.1.1.1.3	447033	Gujarat	Saudi Arabia, UK, Turkey	B.1.36	A2a
			Turkey, Finland, UK		
A1.6.1.1.1.4	451149	Gujarat	Turkey, Finland, UK	B.1.36	A2a
A1.6.1.1.2	444469	Gujarat	Turkey, Finland, UK	B.1.36	A2a
A1.6.1.2	444456	Gujarat	Turkey, Finland, UK	B.1.36	A2a
A1.6.1.3	444484	Gujarat	Turkey, Finland, UK	B.1.36	A2a
A1.6.1.4	455021	Gujarat	Saudi Arabia, UK, Turkey	B.1.36	A2a
A1.6.1.5	437449	Gujarat	Turkey, Finland, UK	B.1.36	1. A2
					2. A2a
A1.7	436414	1. Assam	India, Singapore, Australia	B.6	A1a
		2. West Bengal			
A1.8	436426	1. Bihar	India, Singapore, Australia	B.6	1. A3
		2. Delhi			2. A7
A1.9	437447	Gujarat	UK, USA, Australia	B.1	A2a
A1.9.1	447549	Gujarat	UK, USA, Australia	B.1	A2a

### Lineage and subtype analysis

We also ascertained the lineage and subtype of the observed sequences through Pangolin and Covidex, respectively. Also, the distribution of lineages present in India across the world was assessed through Pangolin. The fact that phylogenetic lineage of SARS-CoV-2 genomes from India exhibits its incidence in diverse countries such as USA, Australia, UK, Singapore, China, and Turkey is reflective of the global nature of the pandemic. Most of it can be attributed to international air travel and diverse regulations across countries.

The three most common lineages in India as predicted by Pangolin are B6, B1, and B1.36, whereas clade A2a appears to be the most predominant one as predicted by Covidex (Fig 2C and Tables 3 and S4). The prevalence of these lineages can shift with increasing incidences and accumulating variations and hence requires regular monitoring. However, proper recording of both national and international travel history for all the patients will go a long way in unveiling the true path of viral evolution.

Table S4 Details of lineage analysis of studied genomes.

## Discussion

The analysis of SARS-CoV-2 sequences from India through phylo-geo-network was carried out with the intention of analyzing the evolution of SARS-CoV-2 along with the geographical context. Subsequent to the Multiple Sequence Alignment, the network was constructed using 152 PI sites excluding the ambiguous sequences to ensure that only those sites wherein there was no sequence ambiguity formed the basis of the network.

Several observations can be drawn from the phylo-geo-network of the studied genomes as shown in Fig 1. First, the core of the network with maximum genomes (157) is the node of reference sequence of SARS-CoV-2 from Wuhan, China, with NCBI accession number NC_045512.2. The fact that this accounts for over one fourth (25.7%) of the total studied sequences is a clear indication that in spite of many reported variations, the original SARS-CoV-2 genome continues to be the dominantly prevalent form. Though the variations are fast accumulating in the virus, it’s the original one that still prevails, at least in the Indian context. Viral evolution is a dynamic and fast process but unless due selection advantage is offered, a new form would not take over.

Second, the distribution of sequences from across India (Fig 1) do not corroborate with the incidence scenarios but are a reflection of the ground level preparations and activity in getting the genomes sequenced. For instance, the under-representation of Maharashtra and Tamil Nadu in the present data set in-spite of being the two most affected states. However, assuming that the virus has an equal chance of evolving anywhere, we believe the number of sequences analyzed is apt for giving a glimpse of the ongoing viral evolution.

Third, when we analyzed the distribution of PI sites across the genome, we found it to be non-uniform in nature. We studied the distribution in the form of strike-rate of PI sites which we define as the number of bases after which there will be another PI site. This is to say that a region with a strike rate of 20 would mean a PI site every 20 bases and so on. Thus, a lower strike rate will infer a higher density of the PI sites in the region (Table 2). Based on our analysis, the Envelope and Spike protein have a PI strike rate of 45 and 115, respectively (Table 2). Before drawing any conclusions, we need to understand that a higher incidence of PI sites does not necessarily corroborate to driving the evolutionary process as their impact on protein functionality needs to be ascertained first. However, it does indicate the potential genomic regions for the same which herein appear to be Envelope and Spike protein.

The geographical distribution of the haplogroups can be looked at from two different aspects. To begin with, which haplogroup is found in which location. Herein, A1 (NC_045512.2) haplogroup as already mentioned was most widely prevalent with 157 sequences distributed across 16 locations. All other haplogroups had 10 or fewer genomes spread across one to three locations (Fig 2A). The scenario is more interesting if we inverse the analysis as in which location had how many haplogroups. Gujarat with a maximal representation of 199 genomes had 27 different haplogroups but this is not the norm as in more sequences would mean more haplogroups. Delhi (63 genomes, 3 haplogroups), Maharashtra (94 genomes, 9 haplogroups), and West Bengal (40 genomes, 7 haplogroups) exhibit the non-linearity of the same. Also, 41 haplogroups have a single location only led by Gujarat (21); Maharashtra (6); West Bengal, Telangana, and Tamil Nadu (4 each); and Ladakh and Orissa (1 each). Three states, Punjab, Andhra Pradesh, and Kerala, do not have any haplogroup so far. The distribution of haplogroups across states has been shown in Fig 1 and Table S2. The fact that some locations with fewer samples have more haplogroups and most haplogroups are localized exclusively to a single state is a clear indication about the local evolution of viruses. However, because the pandemic is still emerging, the final outcome will be clear only at a later stage.

Of the 611 studied genomes, the 51 haplogroups account for 339 genomes. At this juncture, we would like to note about the sequences left out of haplogroups. They belong to haplotypes which may converge to an existing haplogroup or emerge as a new one as the pandemic progresses. Because of the high mutation rate of viruses and with ever increasing incidence of the diseases the virus is replicating more and more and new polymorphisms are being generated every day. These variations are changing the haplotype and haplogroup profile on a regular basis. The lineage and clade analysis of observed haplogroups was carried out through Pangolin and Covidex to correlate the network with global evolution of SARS-CoV-2. This was performed through Pangolin which analyses the evolution lineages and additionally reports their presence in different areas of the world. The observed common lineages in India (B6, B1, and B1.36) and clade A2a as per present sequence congregation needs to be monitored regularly to understand the ongoing viral evolution.

### Conclusions

India provides for a good platform to understanding the emergence and evolution of SARS-CoV-2 pandemic because of its diseases burden spread over a huge and diverse population. The strain most prevalent in India is of the same haplogroup as the SARS-CoV-2 reference sequence from Wuhan indicating absence of any significant novel emerging strain so far. A total of 51 haplogroups have been reported. Geographical distribution of haplogroups across states and the corresponding number of genomes from the state suggest for a local evolution of the virus. The two most common lineages are B6 and B1 whereas clade A2a appears to be the most predominant one in Indian context. A regular update of the sequences and variations therein will help in deciphering SARS-CoV-2 evolution in India.

## Materials and Methods

### Sequence acquisition

Genome sequences of SARS-CoV-2 in FASTA format was assessed from the EpiCov repository (www.epicov.org) of GISAID initiative (Shu & McCauley, 2017) and reference sequence from Wuhan with accession number NC_045512.2 was retrieved from NCBI (www.ncbi.nlm.nih.gov).

**Figure 3.**
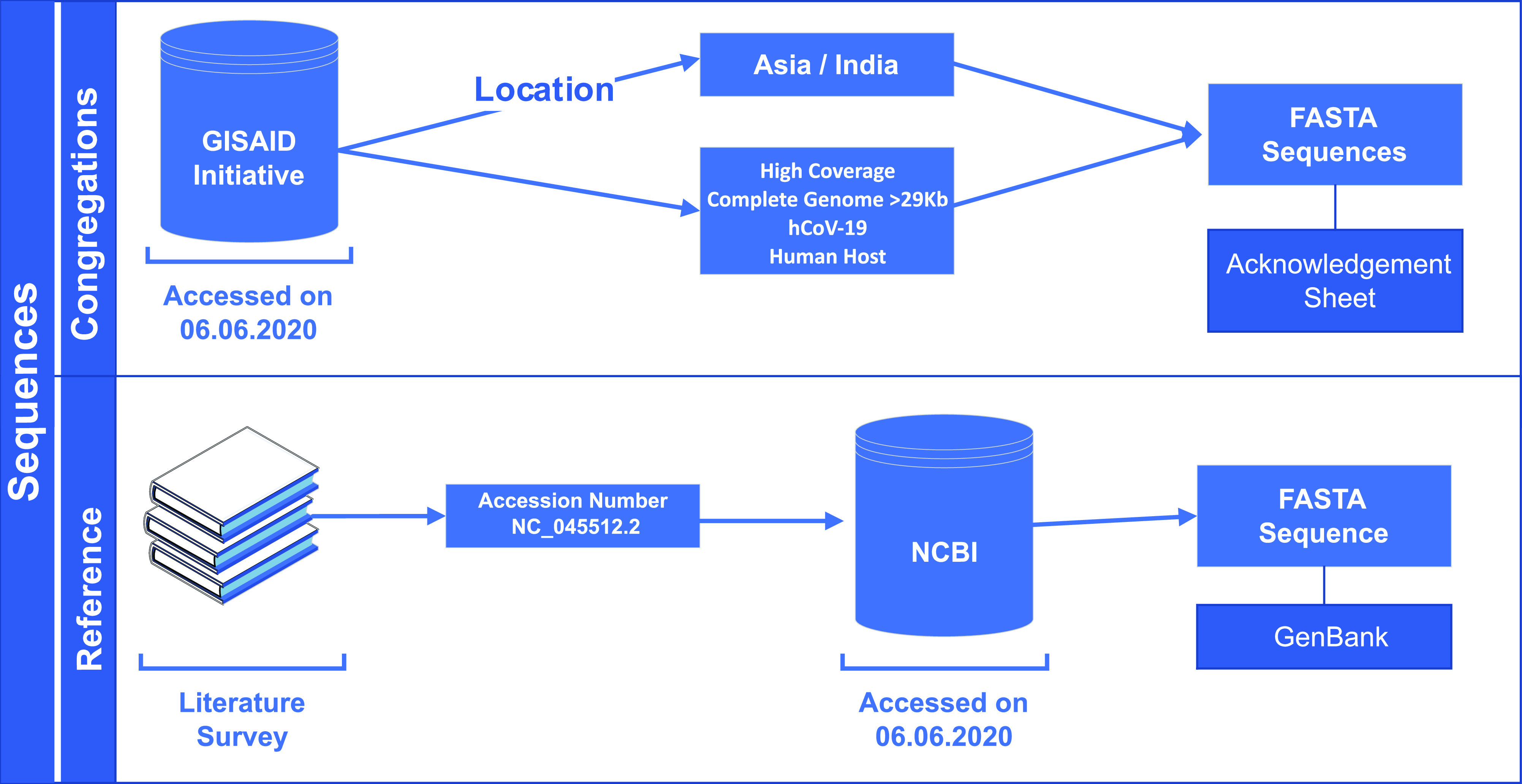
Outline for selection and extraction of sequences used in the study.

On 6 June, 2020, we retrieved 611 FASTA sequence congregations along their rational meta data from GISAID EpiCoV server using the data filter ∼ virus name: hCoV-19 - Host: Human - Location: Asia/India – Complete – High Coverage and use the genome ID by excluding the first part i.e., “EPI_ISL_” of GISAID accession ID. Details of the geographical distribution of the sequences and their accession numbers are provided in Fig 1 and Table S1, respectively. Location data of GISAID are used to identify the state of origin in India, and wherein state name is unavailable, state address of the originating laboratory has been used. The workflow for the acquisition of sequences has been shown in Fig 3.

### Sequence alignment

The congregations are aligned with the FFT-NS-fragment method using rapid calculation of full-length Multiple Sequence Alignment of closely related viral genomes, a light-weight algorithm of Multiple Alignment using Fast Fourier Transform v7 Web server (https://mafft.cbrc.jp/alignment/software/closelyrelatedviralgenomes.html) (Katoh et al, 2018) and keeping alignment size exactly throughout the reference sequence. The nucleotide transformation sites of the alignment were further studied using Molecular Evolutionary Genetics Analysis X (Kumar et al, 2018).

### Phylogenetic network analysis

Aligned sequences were used to generated parsimony based Transitive Consistency Score networks (Clement et al, 2002) implemented in Population Analysis with Reticulate Trees (PopART v1.7) software (Leigh & Bryant, 2015) where more than 5 percent sites contain undefined states and will be masked. A map of haplotypes was also drawn using the same software with geotags and traits label coding.

### Genome annotation

The tool Incorporation of Gene Location in SSR File (IGLSF) (Alam et al, 2019) arranges the location of variable sites according to genes. Using the software DNAPlotter (Carver et al, 2009), we used the Artemis (Carver et al, 2012) to annotate the genome and visualized it as a circular plot.

### Lineage and subtyping analysis

The global lineage to which the identified haplogroups from the sequence congregation belonged was ascertained through Pangolin (Phylogenetic Assignment of Named Global Outbreak Lineages) Web (https://pangolin.cog-uk.io/), using nomenclature implemented by Rambaut et al (2020) *Preprint*. Furthermore, the viral subtypes of the studied genomes from the Indian population was checked using “SARS Cov 2 Nextstrain” classification model of Covidex (https://cacciabue.shinyapps.io/shiny2/), a Web-based subtyping tool (Cacciabue et al, 2020 *Preprint*).

### Sequence statistics

Multiple metrics were used to assess the population genetics to decipher the phylogenetic relationship. We calculated Tajima’s D (Tajima, 1989) statistic to test mutation–drift equilibrium and Π value, segregating sites, parsimony-informative sites to measure DNA polymorphism among sequences using PopART statistics (Leigh & Bryant, 2015).

## Data Availability

All data pertaining to the study has been provided as Supplementary Material of the manuscript.

## Supplementary Material

Reviewer comments
